# An Atypical Case of Oral Squamous Cell Carcinoma of Mandibular Alveolus

**DOI:** 10.1155/2019/2521685

**Published:** 2019-11-13

**Authors:** Seba Abraham, Bhagyalekshmi Mallika, Arunima Reshma, ReejaMol Mohammed Kassim

**Affiliations:** PMS College of Dental Science and Research, India

## Abstract

Oral squamous cell carcinoma is the most common type of oral malignant neoplasm. As per literature, squamous cell carcinomas of the alveolar ridge account for 9% of all the oral carcinomas. The oral squamous cell carcinoma shares clinical similarity with various forms of inflammatory gingival lesions and is often misdiagnosed in our routine dental practice. The dentist should have precise knowledge regarding the clinical manifestation of this deadly disease as early diagnosis and prompt treatment can reduce morbidity and mortality of the disease.

## 1. Introduction

Oral cancer accounts for the eleventh most common cancer worldwide [1]. Oral squamous cell carcinoma (OSCC) is the common oral carcinoma with varied clinical presentations. It accounts for more than 90% of all malignant lesions in the oral cavity [2]. The affected population group was <40 years of age in high-incidence countries such as India, Pakistan, and Sri Lanka [3]. Alveolar ridge SSC accounts for 9% of all cases with oral SCC [4]. Alveolar ridge SSC accounts for the second position, with the first being carcinoma of the tongue as per site specificity. As per local recurrence rate by site, mandibular alveolus carcinoma has the highest local recurrence rate (26/42), second being carcinoma of the tongue (20/47). Squamous cell carcinomas can occur in all areas of the body, but they are most common in the skin and oral cavity.

Oral squamous cell carcinoma of the mandibular region is found to have the lowest survival rate when compared with all other oral carcinomas [5]. Various aids used for assessing mandibular invasion include clinical examination, radiographic examination (OPG, CT dentascan, MRI, bone scans, ultrasound, and PET CT), and histopathological examination. Histopathologic analysis of squamous cell carcinoma reveals two basic patterns of tumor invasion: invasive pattern (infiltrative pattern) and erosive pattern (compressive patterns). In the invasive pattern, islands of tumor infiltrate cancellous spaces and have little osteoclastic activity and no intervening connective tissue. However, in the erosive pattern, the tumor advances as a broad front with active osteoclasts separating the tumor from the bone and the connective tissue layer separating the tumor/bone [6].

Marginal resection is considered as the best treatment option if the carcinoma reaches close to the bone without any invasion or with mild invasion [5]. Marginal resection involves the removal of 1 cm of margins from all sides [7]. Nomura et al. in 2005 reported that in case of oral squamous cell carcinoma, the tumor extended to the periosteum in 33%, to the cortical bone in 23%, and to the bone marrow in 9% of the patients who underwent mandibular resection. The remaining 35% of the patients had no evidence of mandibular invasion. The area of bone resorption on preoperative clinical and radiographic examinations often disagreed with the extent of mandibular invasion on histopathologic examination [8].

The clinical presentation of oral squamous cell carcinoma can range from a white plaque to an ulcerated lesion [9]. Malignant lesions in the gingiva may resemble frequently seen inflammatory lesions of the gingiva. Oral carcinomas can often be misdiagnosed as other inflammatory lesions in the oral cavity leading to delay in delivering prompt treatment. Thus, the early diagnosis and treatment of oral carcinoma by health care providers is mandatory for optimum treatment outcome [10]. Treatment of squamous cell carcinoma is mainly a surgical excision and a radical neck dissection in the case of lymph node metastasis. Radiotherapy is considered as adjunct postoperative treatment along with chemotherapy and is a definite treatment of choice in case of advanced stages of cancer.

Oral carcinoma has emerged as a major health problem in India mainly because of the high prevalence of smokeless tobacco use. Oral SCC metastases to regional lymph nodes in the neck and distant metastases are seen in the later stages as diseases progress [11]. Here, we are reporting a nontobacco-related case of SCC of the lower alveolus with atypical presentation of desquamative gingival lesions and often more chance to misdiagnose the case.

## 2. Case Report

A 48-year-old female patient presented to the Department of Periodontics with the chief complaint of acute pain in relation to the lower right back teeth region for the past 2 months. The patient gave a history of antibiotic and analgesic medication for one week after consulting with a nearby hospital. Pain was the throbbing type and continuous in nature, and it aggravates on opening the mouth, during mastication, and while brushing teeth.

General clinical examination revealed normal mouth opening. Multiple tender lymph nodes were noted on the right side of the face and neck region. On palpation, submandibular and cervical lymph nodes were tender and palpable (2 cm in diameter) and were firm and fixed to the skin.

**Figure 1:**
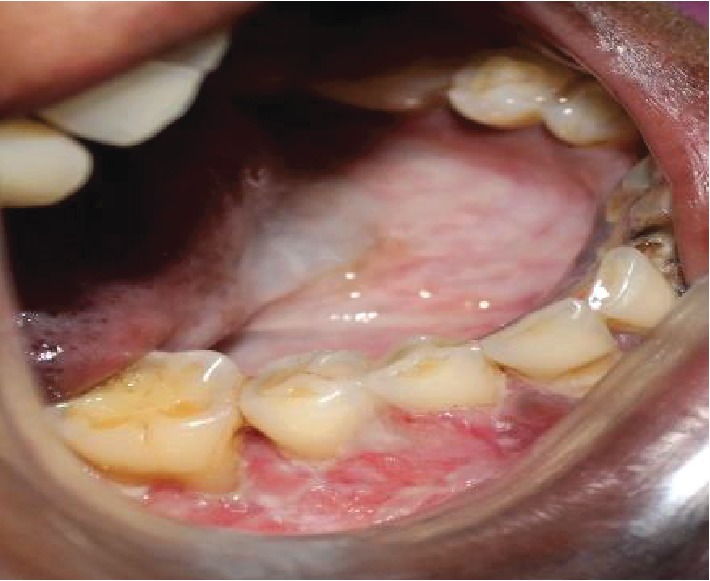
Desquamative changes in relation to the 43, 44, 45, and 46 regions.

**Figure 2:**
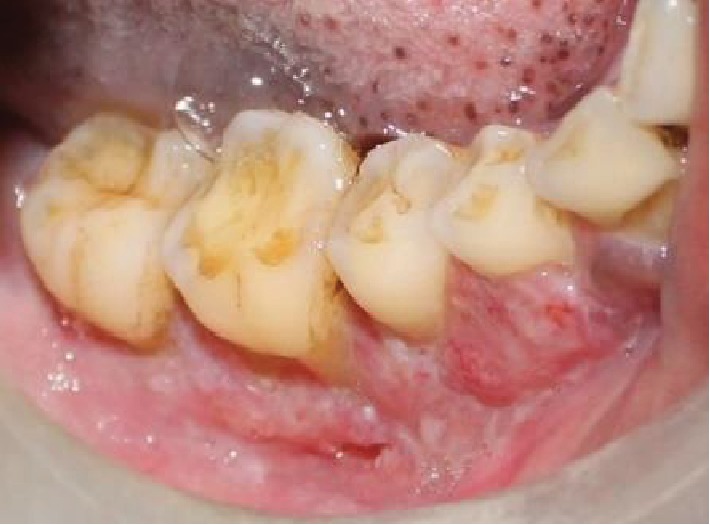
Ulceroproliferative lesion extending from the 44 to 47 regions.

**Figure 3:**
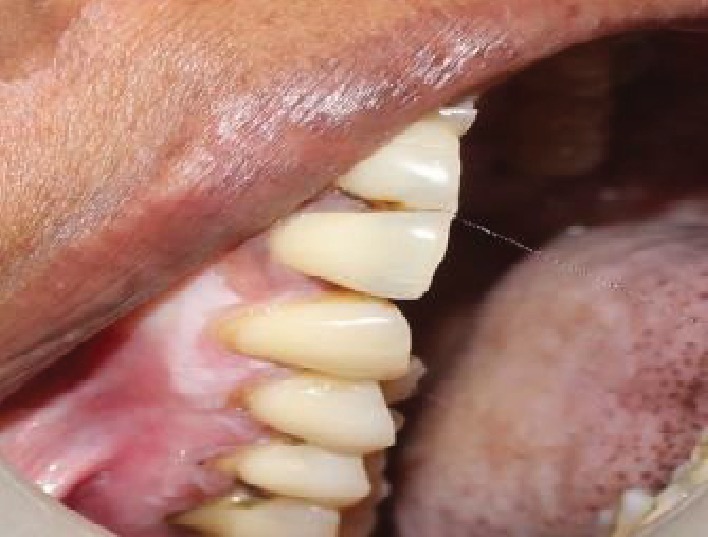
Desquamative lesion noted in relation to 13 extending to the 15 regions.

The intraoral examination revealed desquamative changes in relation to the 43, 44, 45, and 46 regions (Figure 1). Grade 2 mobility in relation to the 44, 46, and 47 regions and grade 3 mobility in relation to 45. No associated paraesthesia was reported. An ulceroproliferative lesion extending from the 44 to 47 regions with mixed keratotic and erythematous area with granular appearance was noted (Figure 2). On palpation, the lesion was tender with mild induration. Desquamative lesion was noted in relation to 13 extending to 15 regions (Figure 3). Periodontal probing resulted in profuse bleeding in relation to 45 and 46 regions. Deep pockets (>6 mm) in relation to the 16, 17, 26, 27, 44, 45, 46, and 47 regions.

Hard tissue examination revealed an end-on-end occlusion. A comprehensive periodontal examination was done which revealed heavy deposits of calculus on the lingual surface of mandibular posteriors. Generalized cervical abrasion and attrition were present. The oral hygiene maintenance was compromised due to severe pain while brushing interfering with proper plaque removal.

### 2.1. Radiographic Evaluation

**Figure 4:**
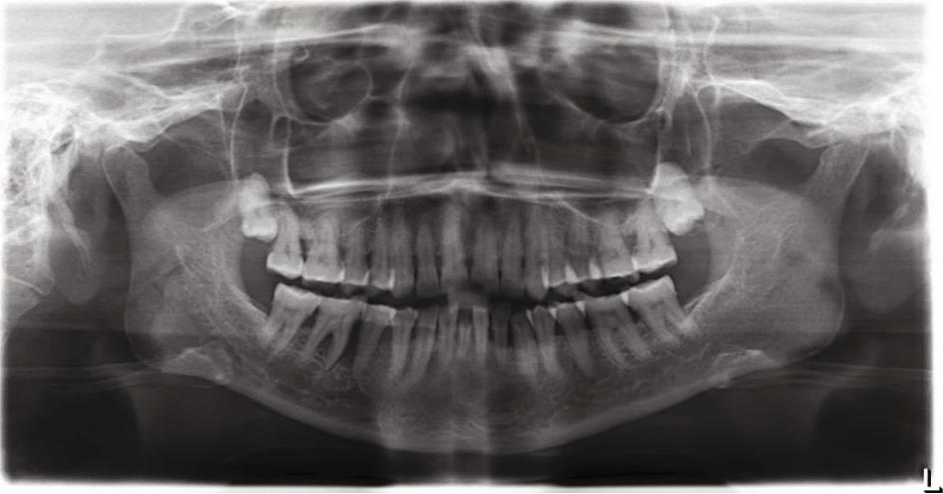
Generalized horizontal bone loss with severe bone loss in relation to the 45 and 46 regions with widening of the PDL space and furcation involvement in relation to 46. Impacted 18 and 28.

**Figure 5:**
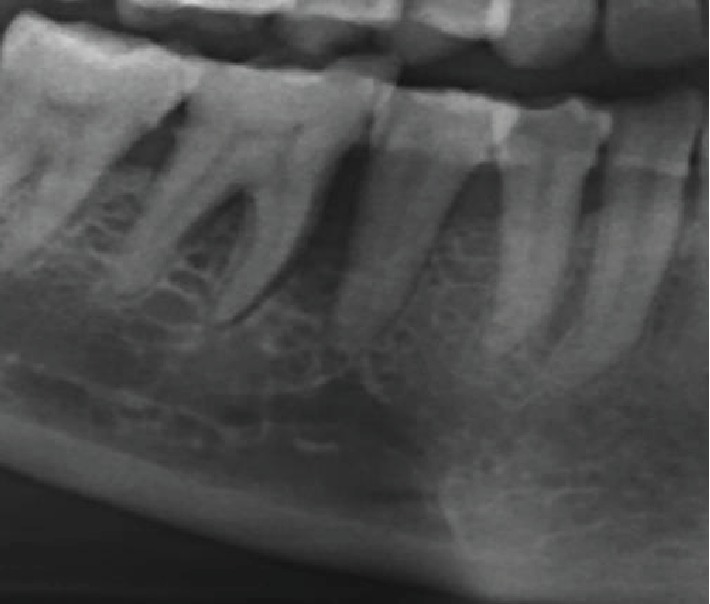
Diffused poorly defined radiolucency was noted in relation to 45 and 46 extending up to the superior border of the mandibular canal.

OPG revealed generalized horizontal bone loss with severe bone loss in relation to the 45 and 46 regions with widening of PDL space and furcation radiolucency in relation to 46. Impacted 18 and 28 were noted (Figure 4). Diffused poorly defined radiolucency was noted in relation to 45 and 46 extending up to the superior border of the mandibular canal (Figure 5).

Based on clinical and radiographic examination, a provisional diagnosis of chronic generalized periodontitis and carcinoma alveolus was made. Differential diagnosis is erosive lichen planus.

Staging of oral squamous cell carcinoma is done based on the American Joint Committee on Cancer. cTNM is the stage given after the clinical examination of the patient, while pTNM is the stage after the histopathological examination of the surgical specimen. Based on the American Joint Committee on Cancer January 2018, the cTNM cancer staging of this case is AJCC Stage IVA and stage grouping T4aN1M0 as there is involvement to the jaw bone and presence of a palpable lymph node of size 2 cm.

### 2.2. Treatment

**Figure 6:**
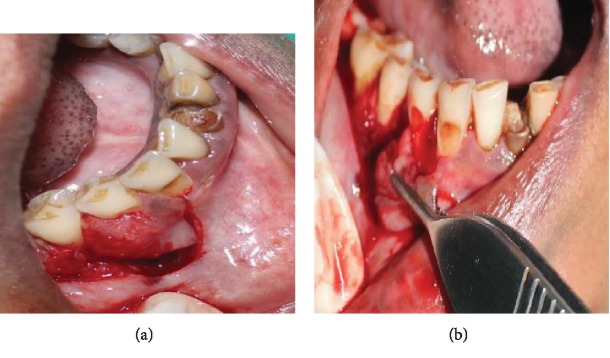
(a, b) Incisional biopsy.

**Figure 7:**
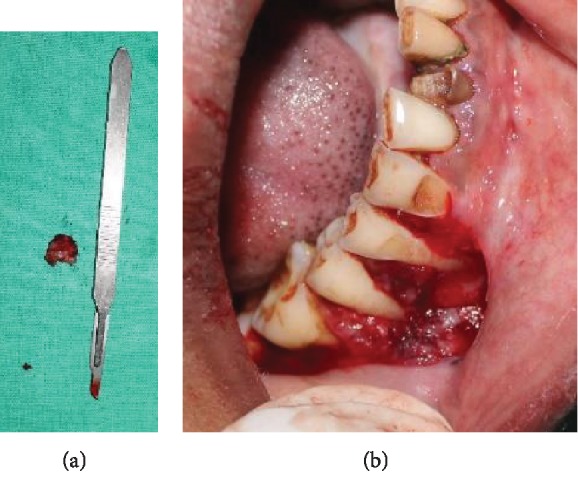
(a, b) Excised tissue and surgical site after excision.

**Figure 8:**
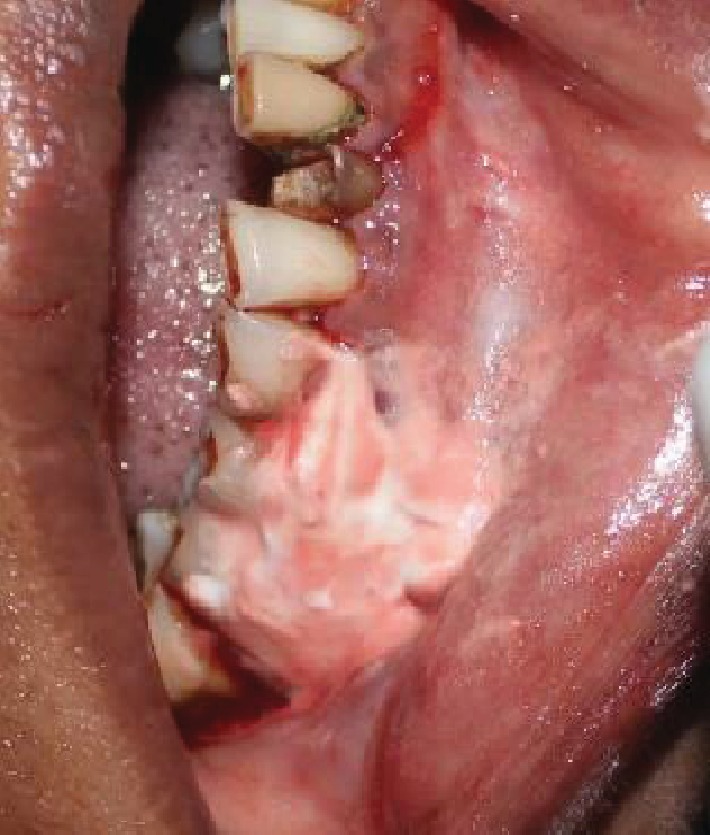
Surgical pack.

Initially, nonsurgical periodontal therapy including scaling and root planing was performed. Incisional biopsy of the oral lesion extending from the marginal gingiva of 44 to 46 to the mucogingival junction (measuring about 1.4∗1.3∗0.6 cm in size) was performed under LA (Figure 6). The excised tissue was sent for histopathological examination (Figure 7). 3-0 vicryl sutures placed and a surgical pack given (Figure 8). The patient was kept under antibiotic and anti-inflammatory therapy for one week.

### 2.3. Histopathological Examination

**Figure 9:**
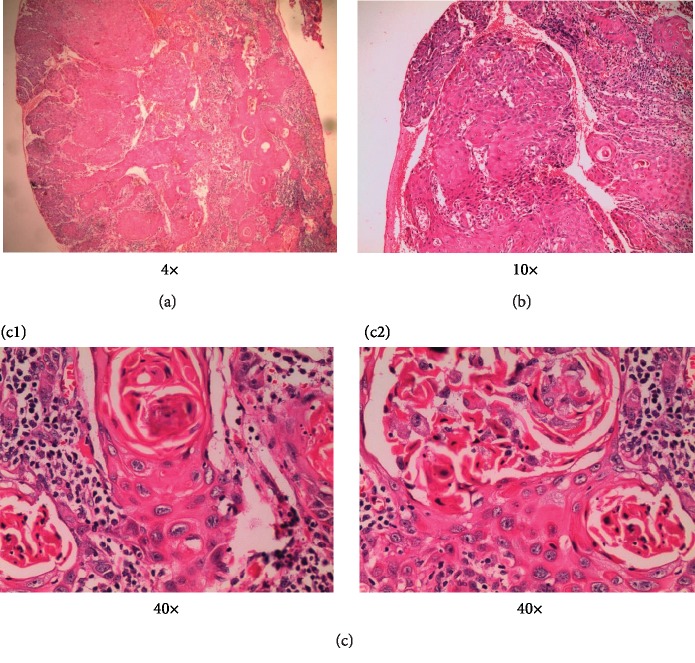
(a) 4x and (b) 10x: histopathologic view of the lesion showing islands and sheets of malignant squamous epithelial cells infiltrating into the connective tissue. Areas showing severely atrophic epithelium with few saw toothed rete pegs and basal cell degeneration are also seen. (c1, c2) 40x: higher magnification showing neoplastic cells with numerous keratin pearl formations, nuclear and cellular pleomorphisms, and mitotic figures. Few dilated blood vessels were also noted.

Histopathological examination of the given soft tissue section revealed islands and sheets of malignant squamous epithelial cells infiltrating into the connective tissue (Figures 9(a) and 9(b)). The neoplastic cells exhibit numerous keratin pearl formation, nuclear and cellular pleomorphism, and mitotic figures (Figure 9(c) (c1, c2) 40x). Areas of severely atrophic epithelium with few saw toothed rete pegs and basal cell degeneration are also seen. Based on clinical, radiographic, and histopathological examinations, the case is diagnosed as squamous cell carcinoma (SCC). The patient was referred to the Regional Cancer Centre (RCC), Thiruvananthapuram, for further treatments. We tried to contact the patient for further follow up but was in vain.

## 3. Discussion

Squamous cell carcinoma is considered as the most common malignant neoplasm of the oral cavity. The tongue, oropharynx, and floor of the mouth are the most common sites, and SCC of the gingiva and lips is rarely seen. SCC of the mandibular gingiva is more common than the maxillary gingiva [12].

Most of the cases of oral carcinoma are associated with tobacco chewing habit and usually appear as a premalignant lesion like leukoplakia before progressing to the malignant stage, but rare cases have also been reported with nontobacco-associated squamous cell carcinoma. The case reported here is one without the history of tobacco chewing habit.

Carcinoma of the gingival region often mimics desquamative lesions of the gingiva and other inflammatory gingival lesions. The gingiva is one of the most common sites for chronic inflammation as it is often associated with irritants like calculus and abundant microbial flora. Attached gingiva is more involved than free gingiva [4]. Misdiagnosis is often encountered in the absence of detailed clinical examination and radiographic investigation.

Mandibular alveolus is the second most common site for oral carcinoma. Oral squamous cell carcinoma is more frequently seen among men compared to women as men are often exposed to high risk habits such as smoking and tobacco chewing (Liviu Feller et al. 2012). Age is another critical factor for oral SCC; as age advances, pronounced genetic and epigenetic changes take place. The case presented here is a female patient of age 48 years with a lesion mimicking desquamative lesion in relation to the lower right back teeth region. Another desquamative lesion was noted in the maxillary region in relation to 13, 14, and 15 without the clinical presentation of SCC.

Regional lymph node metastasis is another feature of squamous cell carcinoma. Cervical lymph nodes of the submandibular triangle and upper jugular regions have stronger predilection of regional lymph node metastasis in the case of SCC of the lower alveolus [13]. Tender and palpable submandibular and cervical lymph nodes (which are firm and fixed to the skin) of about 2 cm in diameter were detected in the present case. Prognosis is better in early oral SCC, especially those that are well-differentiated and without metastasis, but the worst part is that most cases of OSCC are not diagnosed at the earlier stage of the disease. The prognosis can vary based on a number of factors that are related to the tumor or treatment or to the patient.

Squamous cell carcinoma was found to enter the medullary cavity through the upper border of the mandible, either through the occlusal ridge alone or in combination with penetration of either the buccal or lingual plates. The spread through the foramina is another important route of entry. When the carcinoma is not that deep, that is, if it does not reach the alveolar canal, then there is no spread along the alveolar canal and little or no insinuation of the cancer cells beyond the tumor front [7].

The radiologically detected bone defects in squamous cell carcinoma were classified as follows: (a) erosive—well-defined margins of the absorbed bone and (b) moth eaten—irregular, ill-defined margins of absorbed bone. Histopathologic patterns of bone involvement were classified as follows: (a) expansive—outline of the eroded bone appears smooth or slightly concave and (b) infiltrative—tumors will infiltrate the mandible through spaces or aggressively by destroying the bone. The extent of radiologically detected bone defects and the histological pattern of bone involvement can be graded as follows: (a) grade 1—bone defects limited to the alveolar bone, (b) grade 2—bone defects exceeded the alveolar bone but did not extent beyond the mandibular canal or the temporary line between right and left mental foramen, and (c) grade 3—bone defects extend beyond the mandibular canal or the temporary line between the right and the left mental foramen [14]. The case presented here shows diffuse, ill-defined radiolucency in relation to 45 and 46 extending up to the superior border of the mandibular canal.

Treatment of squamous cell carcinoma is primarily a surgical excision followed by radiation therapy and chemotherapy as postoperative adjunct treatment modalities. Radical neck dissection is often required in case of lymph node metastasis. Marginal resection is considered as a treatment option when the bone defects did not extend beyond the mandibular canal and segmental resection if it extends beyond the mandibular canal. The 5-year cumulative survival rate for the mandibular marginal resection group is about 78.1% and 72.8% in the segmental resection group [14]. Other innovations in the field of cancer therapy were laser-based technology (photodynamic therapy), immunotherapy, and gene therapy to treat oral squamous cell carcinoma at a much earlier stage [15].

## 4. Conclusion

Squamous cell carcinoma is the most common malignant epithelial neoplasm with varied oral presentations. Therefore, the dentist should be aware of the characteristics of the disease. The most fatal complication is the distant metastasis as the disease progresses. Hence, correct and timely diagnosis is of utmost importance and there are more chances for misdiagnosis as the clinical presentation of oral squamous cell carcinoma can mimic inflammatory gingival lesions.
